# Mental Health Problems during the COVID-19 Pandemics and the Mitigation Effects of Exercise: A Longitudinal Study of College Students in China

**DOI:** 10.3390/ijerph17103722

**Published:** 2020-05-25

**Authors:** Yao Zhang, Haoyu Zhang, Xindong Ma, Qian Di

**Affiliations:** 1 Division of Sports Science & Physical Education, Tsinghua University, Beijing 10084, China; yao-zhan19@mails.tsinghua.edu.cn (Y.Z.); maxd@tsinghua.edu.cn (X.M.); 2 School of Sport Business, Beijing Sport University, Beijing 10084, China; helenmay7@163.com; 3 Research Centre for Public Health, Tsinghua University, Beijing 10084, China; 4 Vanke School of Public Health, Tsinghua University, Beijing 100084, China

**Keywords:** coronavirus, mental health, sleep quality, physical activity, mitigation strategies

## Abstract

(1) Background: The novel coronavirus disease 2019 (COVID-19) is a global public health emergency that has caused worldwide concern. Vast resources have been allocated to control the pandemic and treat patients. However, little attention has been paid to the adverse impact on mental health or effective mitigation strategies to improve mental health. (2) Purpose: The aim of this study was to assess the adverse impact of the COVID-19 outbreak on Chinese college students’ mental health, understand the underlying mechanisms, and explore feasible mitigation strategies. (3) Methods: During the peak time of the COVID-19 outbreak in China, we conducted longitudinal surveys of sixty-six college students. Structured questionnaires collected information on demographics, physical activity, negative emotions, sleep quality, and aggressiveness level. A mixed-effect model was used to evaluate associations between variables, and the mediating effect of sleep quality was further explored. A generalized additive model was used to determine the dose-response relationships between the COVID-19 death count, physical activity, and negative emotions. (4) Results: The COVID-19 death count showed a direct negative impact on general sleep quality (β = 1.37, 95% confidence interval [95% CI]: 0.55, 2.19) and reduced aggressiveness (β = −6.57, 95% CI: −12.78, −0.36). In contrast, the COVID-19 death count imposed not a direct but an indirect impact on general negative emotions (indirect effect (IE) = 0.81, *p* = 0.012), stress (IE = 0.40, *p* < 0.001), and anxiety (IE = 0.27, *p* = 0.004) with sleep quality as a mediator. Moreover, physical activity directly alleviated general negative emotions (β = −0.12, 95% CI: −0.22, −0.01), and the maximal mitigation effect occurred when weekly physical activity was about 2500 METs. (5) Conclusions: (a) The severity of the COVID-19 outbreak has an indirect effect on negative emotions by affecting sleep quality. (b) A possible mitigation strategy for improving mental health includes taking suitable amounts of daily physical activity and sleeping well. (c) The COVID-19 outbreak has reduced people’s aggressiveness, probably by making people realize the fragility and preciousness of life.

## 1. Introduction

The novel coronavirus disease 2019 (COVID-19) has swept across the world, causing a global pandemic [1]. On 30 January 2020, the World Health Organization (WHO) declared COVID-19 an international public health emergency. Until April 15, 2020, nearly 2 million confirmed COVID-19 cases, including 123,010 deaths, have been reported worldwide. With respect to the COVID-19 outbreak in China, Wuhan, the first place to report the COVID-19 disease, was placed in lockdown status on January 23, 2020 by the Chinese government, and the whole country then entered level-1 emergency status. During the period from January 23 to March 20, 2020, the cumulatively confirmed infection and death counts of COVID-19 in China increased to over 80,000 and around 3500, respectively, and still present an increasing trend.

Recently, researchers have spared no efforts to understand the clinical features of COVID-19, prevent the disease from spreading, and develop vaccines. Few studies have focused on its adverse impact on mental health [2]. A previous study observed a neuropsychiatric linkage between the outbreak of acute emergency and mental distress, not only in sufferers but also in the general population, which has led to long-term social unrest [3]. Similarly, in the aftermath of a human-made disaster, the terrorist attacks of September 11, 2011, 20% of respondents who lived near the World Trade Centre reported post-traumatic stress disorder (PTSD) and 9.7% reported depression symptoms, indicating considerable psychological morbidity in the general population [4]. Similar mental disorder symptoms were observed in different groups of people in response to acute natural disasters, such as earthquakes [5] and hurricanes [6]. Moreover, previous studies of Middle East respiratory syndrome coronavirus (MERS) [7,8] and severe acute respiratory syndrome (SARS) [9,10] have shown increased psychiatric comorbidities, including depression, anxiety, panic attack, and suicidality.

During the period of the COVID-19 pandemic, all healthy residents, especially college students, are recommended to maintain physical distancing [11]. However, this may result in cognitive distress, negative emotions, and aggressiveness due to the fear of COVID-19, reduced sleep quality, or a sense of numbness. According to recent surveys, nearly 24.9% of college students in China reported anxiety symptoms [12]. Similarly, 53.8% of Chinese residents rated the psychological impact of the COVID-19 outbreak at moderate or severe levels, including 16.5% with depressive symptoms and 28.8% with anxiety [11]. However, these cross-sectional surveys do not provide sufficient evidence on the relationship between the COVID-19 outbreak and adverse mental health status or aggressiveness.

Although some experts have advanced possible measures to mitigate widespread negative emotions, such as increasing telephone psychiatric treatment [13,14], they are not long-term plans that take into consideration the increased social burden and medical pressure. In fact, many previous studies found that mental health is associated with physical activity, sleep quality, and quality of life in both clinical and nonclinical populations [15,16]. Individuals with increasing physical activity can reduce mental illness morbidities by improving sleep quality [17]. Likewise, some home or indoor exercises, such as high-intensity interval training (HIIT) and relaxing training, also showed benefits to physical and mental health [18,19]. However, few studies have explored the possibility of improving the mental health of ordinary people during the COVID-19 outbreak.

Therefore, in this study, we surveyed the mental health status affected by the COVID-19 outbreak among college students in China. This longitudinal study has four purposes: (1) to provide longitudinal evidence on the relationship between the COVID-19 outbreak and mental health as well as aggressive behaviours; (2) to explore the underlying mechanism whereby COVID-19 negatively affected emotions during the peak time of the outbreak; (3) to examine the mitigation effects of exercise on mental disorders; and (4) to determine the dose-response relationship between physical activity and mental distress. The present study may assist policymakers and healthcare professionals in conducting effective psychological interventions and advancing cost-effective suggestions to avoid negative emotions for general residents when isolated at home.

## 2. Materials and Methods

### 2.1. Designs and Participants

We adopted a longitudinal survey design to assess the negative emotions, sleep quality, aggressiveness, and physical activity among Chinese college students who were forced to stay at home during the COVID-19 outbreak using three consecutive online questionnaires. Before the online surveys, we posted recruitment information for the longitudinal surveys on the internet via WeChat moments and WeChat pushes. Since we intended to explore the mitigation effects of exercise on negative emotions, the participation selection criteria in this study were that college students, who stayed at home in a social distancing status, should be physically healthy and non-disabled. After seeing the recruitment information, sixty-six college students from several provinces participated voluntarily in the survey. All of them met the enrolled criteria, and none of them were diagnosed with COVID-19 disease by the end of the online survey. As per national policy, participants stayed at home to implement physical distancing to stop disease transmission.

They were instructed to complete the online questionnaires every half month, and the three examined times were 9:00 a.m.–12:00 p.m. on February 19, 2020, 9:00 a.m.–12:00 p.m. on March 5, 2020, and 9:00 a.m.–12:00 p.m. on March 20, 2020. The language of all the online questionnaires was Chinese, and all the questionnaires used in this study have been validated among Chinese populations in previous studies. Participants were asked to complete and submit the online questionnaires on time. Ethical approval was obtained from the Institutional Review Board of Tsinghua University (ID number: 20190091). Detailed information about this study was posted at the top of the questionnaires. Survey participants were asked to sign a consent form before completing the structured questionnaire each time. A total of 70 Yuan (equivalent to 10 dollars) was offered to each participant upon receiving all completed questionnaires.

Before the longitudinal surveys, a total of 66 college students were invited to this study. During the investigations, 66 participants who volunteered to participate in this study completed the online questionnaires twice, and 59 of them completed the surveys three times. In order to explore longitudinal evidence, the survey data for participants who filled online questionnaires at least twice were used to analyze. Hence, the final sample size included in the study analysis was 66 participants.

### 2.2. Measurements

#### 2.2.1. Physical Activity

Information on physical activity (PA) was collected via the short version of the International Physical Activity Questionnaire (IPAQ-S), which is suitable for people aged between 15 and 69 years and is primarily used for population surveillance of physical activity levels. The IPAQ-S (7 items) has been validated in Chinese with good reliability [20,21]. Participants were asked to classify their weekly PA during the recent half month into three categories: light, moderate, and vigorous. Total minutes of vigorous physical activity in the last week were constructed based on two questions: “During the last seven days, how many days did you spend on vigorous physical activities like lifting, fast cycling, and fast swimming?” and “How much time did you usually spend each time on these vigorous physical activities?” Total minutes of vigorous PA were computed by multiplying the daily average minutes of vigorous PA by the corresponding intensity days. Likewise, the total minutes of moderate and light PA in the last week were calculated in the same way. According to Ainsworth et al. [22], the ratio of work metabolic rate to a standard resting metabolic rate (MET) was used to estimate the weekly energy expenditure. Energy expenditure in MET-minutes per week (METs) can be estimated for specific activities by type and intensity [22,23]. Referring to previous studies [22,23], the average MET coefficients of light, moderate, and vigorous PA in IPAQ-S are 3.3, 4, and 8, respectively. The final light, moderate, and vigorous physical activity were computed by multiplying the corresponding MET coefficient with the total minutes (e.g., Moderate MET minutes/week (METs) = 4.0 × moderate-intensity activity minutes × moderate days). The METs of total PA were calculated by adding vigorous PA, moderate PA, and light PA. Sedentary behavior was defined as PA less than 1.5 METs [22,23].

#### 2.2.2. Sleep Quality

Subjective sleep quality was measured using the widely used Pittsburgh Sleep Quality Index (PSQI), which assesses the overall weekly sleep quality in the last month [24]. We used 19 self-rated items of the validated Chinese version of the PSQI (C-PSQI) in this study [25]. The C-PSQI used in this study includes seven sleep components: subjective sleep quality, sleep latency, sleep duration, sleep efficiency, sleep disturbances, use of sleep medication, and daytime dysfunction. Each component was rated on a scale of 0–3. The global PSQI score, ranging from 0 to 21, is the sum of all the component scores, and a higher global PSQI score indicates poorer sleep quality. According to a previous study, global PSQI with a score greater than 5 scores indicates some degree of poor sleep disorder [26].

#### 2.2.3. Negative Emotions

Negative emotions and mental health during the last week were measured using the Depression Anxiety Stress Scale, with 21 self-reported items (DASS-21) [27], which has been validated in the Chinese population [28,29]. This structured questionnaire was previously used to assess the immediate and sustained psychological distress of healthcare workers during the SARS period [30]. The DASS-21 consists of three components of stress, anxiety, and depression, each of which includes seven items. In terms of stress, participants were asked to answer questions like “I found myself getting quite upset by trivial things.” With regard to depression and anxiety, participants were asked to answer questions such as “I felt that life was not worthwhile” and “I found myself in a situation which made me so anxious and I was most relieved when they ended.” Each item was rated on a scale of 0–3, corresponding to “totally disagree,” “partially agree,” “mostly agree,” and “totally agree.” The score of each component was calculated as the sum of scores for seven related items, with values ranging from 0 to 21. The global DASS score is the sum of the three components and is used as a general indicator of mental distress. The score for each component ranges from 0 to 21, and the global DASS score, which the sum of the three components, serves as a general indicator of mental distress. In addition, higher scores of stress, anxiety, depression, and global DASS represent more serious negative emotions. Scores greater than 10, 7, and 9 on stress, anxiety, and depression, respectively, may indicate significant levels of the corresponding negative emotions [31].

#### 2.2.4. Aggressiveness

Aggressiveness was measured using the Buss-Perry Aggressive Questionnaire (BPAQ), which assesses aggressive emotions, intentions, or behaviors [32,33]. The BPAQ has been demonstrated to be a reliable measure of aggressiveness in the Chinese population [34,35]. The BPAQ consists of four components: physical aggression, verbal aggression, anger, and hostility, with 5, 6, 3, and 8 items, respectively. Each item was rated on a scale of 1 (*totally disagree*) to 5 (*totally agree*). The score of each component was the sum of scores for the corresponding items, and the overall aggressiveness score was the sum of the four components. A higher score indicates a higher aggressive level.

#### 2.2.5. COVID-19 Data

The COVID-19 disease data were extracted from the open-source git-hub packages “canghailan/Wuhan-2019-nCoV” and “GuangchuangYu/nCoV2019,” which comprise cumulative numbers of confirmed, suspected, cured, and death cases at the provincial level from January 1, 2019, to the present day. We extracted the number of cumulative death cases one day before as the indicator of COVID-19 outbreak severity for each province and then matched provincial-level COVID-19 death cases to each participant based on home address.

### 2.3. Statistical Analysis

Considering longitudinal measurements and inter-person variation, we employed a mixed-effect model with a random effect on individuals to examine the relationship between COVID-19 death cases, sleep quality, physical activity, negative emotions, and aggressiveness. Variables that did not change over time were controlled using this analytic approach. We used the mediation package of R software to explore the mediation effect of sleep quality on the association between COVID-19 death count and negative emotions in order to discover the possible underlying influence mechanism. In addition, we examined the mitigating effects of PA on negative emotions and then explored the dose-response relationship between the global DASS score, COVID-19 death count, and physical activity by fitting a generalized additive model with splines. Statistical analysis was performed using R software, version 3.6.3 (R Project for Statistical Computing) and a two-tailed *p* < 0.05 was considered statistically significant.

## 3. Results

### 3.1. Characteristics of the Survey Participants

A summary of participants’ demographic information and health behaviors at baseline is shown in Table 1. A majority of the sample comprised female participants (62.12%) and most participants were around 20 years old (20.70 ± 2.11), and only a few of the participants were from non-Han minorities. Most of the participants lived in urban areas during the COVID-19 outbreak. With regard to health behaviors, participants exerted 354.55 METs (*SD* = 613.41) of vigorous physical activity every week on average. The amount of physical activity was significantly higher in male participants than female ones. Likewise, male participants consumed more energy in light PA (*p* = 0.005), vigorous PA (*p* < 0.001), and total PA (*p* < 0.001) than did female participants. However, no significant differences were observed in terms of moderate PA and total minutes of weekly sedentary behavior, and no gender difference was observed in the groups with good sleep quality (PSQI ≤ 5) and poor sleep quality (PSQI > 5). It is worth mentioning that nearly 85% of respondents reported worries or concerns about COVID-19 disease, and 28.79%, 45.45%, and 22.73% of them reported stress, anxiety, and depression emotions. In addition, the percentage of female young adults reporting abnormal levels of every negative emotion was higher than in male respondents, even though the differences were not statistically significant. Moreover, all enrolled participants were non-smokers according to the demographic information. This may be because all of the participants were college students and the sample size in this study was not too big.

### 3.2. Epidemic Trends of the COVID-19 in China from January 23 to March 20, 2020

**Figure 1:**
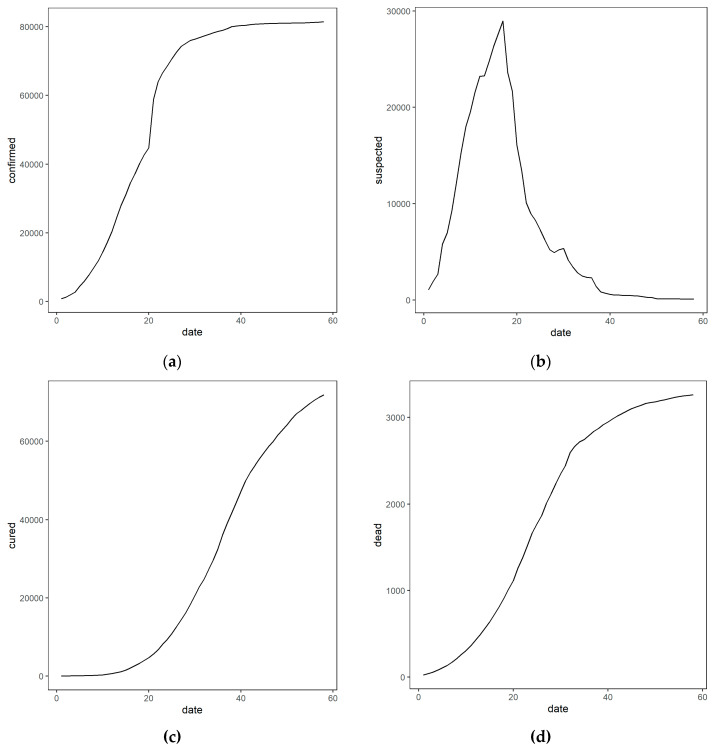
The national epidemic trend of the COVID-19 in China from January 23 to March 20, 2020. Note: The subgraph of (**a**–**d**) refer to the relationship between date and cumulative confirmed, suspected, cured, and death counts, respectively. The Chinese government declared a lockdown in Wuhan on January 23, which then entered the level-1 emergency response. All provinces across China entered a state of blockade. Thus, we regard January 23, 2020 as the starting point (date 1) of our study and a total of 58 days (from date 1 to date 58) were recorded until the last day of the online survey, March 20, 2020.

Figure 1 shows the development trend of the COVID-19 pandemic in China from January 23 to March 20, 2020 for the cumulative numbers of cases of confirmed, suspected, and cured patients and of deaths. The numbers of cumulatively confirmed cases and deaths continued to climb for two months, with a sharp increase in the number of cumulatively confirmed cases in the first month. During the period of repeated online surveys, from February 19 to March 20, 2020, the severity of COVID-19 continued to increase and gradually reached its peak.

### 3.3. Relationships between Variables

Table 2 shows the relationships between COVID-19 deaths and physical activity, along with sleep quality, aggressiveness, and negative emotions. Local COVID-19 death cases were negatively associated with participants’ general sleep quality. Every 1000 increase in local COVID-19 deaths was associated with a rise of 1.37 (95% confidence interval [95% CI]: 0.55, 2.19) units in the global PSQI score, indicating a decline in sleep quality. Among the seven components of PSQI, sleep efficiency was significantly associated with COVID-19 death cases, with a 1000 increase in local death cases corresponding to a 0.29 (95% CI: 0.15. 0.44)-unit increase in sleep efficiency score, indicating reduced sleep efficiency. In addition to sleep efficiency, COVID-19 death cases were also associated with decreased sleep quality and sleep duration and increased sleep disturbance and daytime dysfunction, although these associations were insignificant.

However, negative emotions, global DASS scores, stress, anxiety, and depression did not show any directly significant associations with the COVID-19 death count. In contrast to negative emotions, the aggressiveness score was negatively associated with the local COVID-19 death count. Every 1000 increase in local COVID-19 deaths was significantly associated with a change of −6.57 units (95% CI: −12.78, −0.36) in the aggressiveness score, indicating a declined aggressiveness level. Furthermore, negative emotions can be significantly alleviated by physical activity. Each 100-unit increase in METs of total physical activity corresponded to a change of −0.12 (95% CI: −0.22, −0.010) in the global DASS score. Physical activity also significantly alleviated depression (*p* = 0.040); however, the alleviation effects on stress were insignificant (*p* = 0.090, Table 2). Unlike negative emotions, physical activity, sleep quality, and aggressiveness was not significantly associated.

### 3.4. Underlying Influencing Mechanism

**Figure 2:**
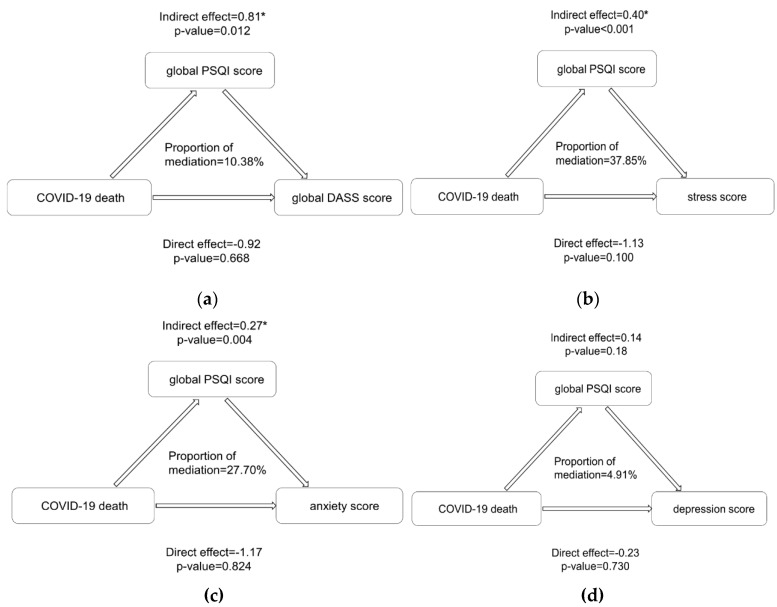
Mediation effects of sleep quality on the relationship between COVID-19 deaths and negative emotions. Note: The subgraph of (**a**–**d**) indicates the indirect influencing path of the COVID-19 on global negative emotions, stress, anxiety and depression, respectively, with sleep quality playing as a mediator. In Figure (**a**), indirect effect = 0.81 means that for every 1000 increase in the COVID-19 death count, there is an indirect effect on the global DASS score and an associated increase in the global DASS score of 0.81, indicating an increased level of negative emotions. The same interpretation also holds for other figures. Asterisks indicate significance at the 5% level. MET: the ratio of work metabolic rate to a standard resting metabolic rate. METs: energy expenditure in MET-minutes per week; PSQI: Pittsburgh Sleep Quality Index. Calculation of mediation effects and proportion of mediation was completed in the mediation package of R software, version 3.6.3.

**Figure 3:**
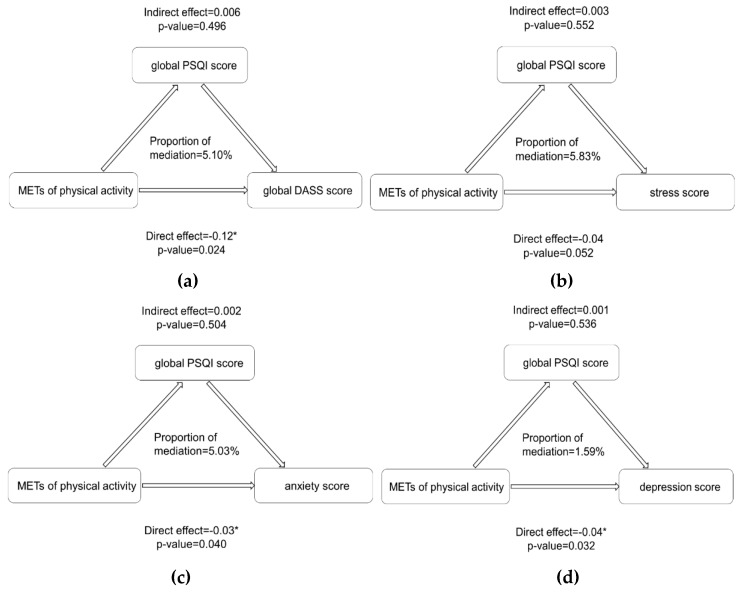
Mediation effects of sleep quality on the relationship between physical activity and negative emotions. Note: The subgraph of (**a**–**d**) indicates the direct influencing path of the physical activity on global negative emotions, stress, anxiety and depression, respectively, without sleep quality playing as a mediator. Asterisks indicate significance at the 5% level. So, no mediation effects were significant in this figure. MET: the ratio of work metabolic rate to a standard resting metabolic rate. METs: energy expenditure in MET-minutes per week; PSQI: Pittsburgh Sleep Quality Index.

Based on the relationships between variables, we further explored the mediation effect of sleep quality on COVID-19 and negative emotions (Figure 2). The effects of the COVID-19 death count on young adults’ stress (indirect effect (IE) = 0.40, *p* < 0.001), anxiety (IE = 0.27, *p* = 0.004), and global DASS score (IE = 0.81, *p* = 0.012) were significantly mediated by decreased sleep quality, and the mediation effect on depression was not significant (*p* = 0.180). However, while physical activity did not show an indirect influence on negative emotions, it did show a direct impact without mediating the effect of sleep quality (Figure 3).

### 3.5. Dose-Response Relationships between Variables

**Figure 4:**
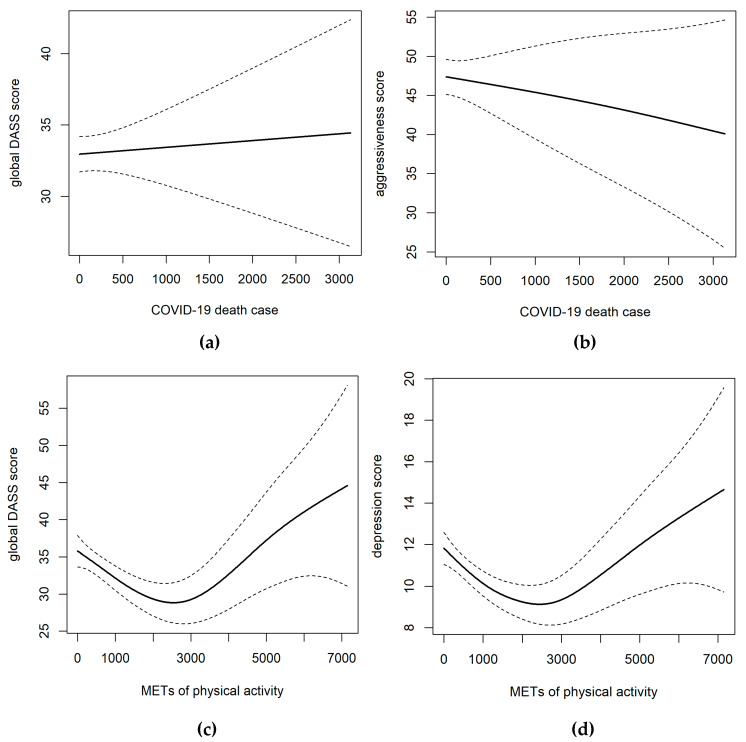
Relationship between COVID-19 deaths, physical activity and negative emotions, and aggressiveness. Note: The subgraph of (**a**–**d**) indicates the relationship between the COVID-19 and global negative emotions, the COVID-19 and aggressiveness, physical activity and global negative emotions, physical activity and depression, respectively. Splines for either the COVID-19 death count or energy expenditure in MET-minutes per week (METs) of physical activity were entered into a mixed-effect model with random effects on individuals. Dashed lines indicate 95% confidence intervals. The global DASS score ranges from 0 to 63, where a larger value means increased negative emotions; depression scores range from 0 to 21, with larger values indicating more depressive emotions; and aggressiveness scores range from 22 to 110, with larger values indicating more potentially aggressive behaviour.

We used a generalized additive model to examine the dose-response associations between the COVID-19 death count, physical activity and aggressiveness, and negative emotions (Figure 4). As Figure 4 shows, the relationship between the COVID-19 death count and negative emotions as well as aggressiveness was linear with no sign of threshold or plateau, although the trends were different. Moreover, the dose-response curve between negative emotions and physical activity exhibited a U-shaped relationship, indicating that either an inadequate or excessive amount of physical activity worsened negative emotions. A suitable amount to minimize negative emotions occurred when weekly physical activity was about 2500 METs, corresponding to 108 min of light, 80 min of moderate, or 45 min of vigorous physical activity every day.

## 4. Discussion

The focus of this longitudinal study is twofold: (1) To investigate the impact of the COVID-19 severity on Chinese college students’ mental health and life status and explore the underlying mechanisms of this effect during the peak time of the COVID-19, from February 19 to March 20, 2020; and (2) to assess the mitigation effects of exercise on negative emotions and advance a suitable physical activity level as a psychological intervention strategy to improve mental health. Our findings suggest that the severity of the COVID-19 outbreak could significantly increase people’s negative emotions through declining sleep quality. In additional, maintaining regular exercise was helpful to alleviate negative emotions, and 2500 METs of PA every week was the optimal load.

### 4.1. Prevalence of Negative Emotions during the Peak Time of the COVID-19 Outbreak

In this study, nearly 85% of respondents reported their worries about COVID-19, and over 20% reported at least one form of mental distress in line with previous acute emergencies [7,8,9]. The prevalence of negative emotions, especially anxious emotions, was higher in this study than in previous studies, which were conducted mainly among Chinese university students in the initial phase of COVID-19 [11,12]. The development trend of negative emotions may imply that the adverse impact of COVID-19 on public mental health will continue to increase as COVID-19 spreads across the world. Furthermore, females suffered a greater psychological impact from the COVID-19 outbreak, with higher but insignificant scores in stress, anxiety, and depression. The lack of significant results probably reflects the limited sample size. This finding is in line with previous epidemiological studies, which found that women were more vulnerable to developing depression or PTSD [36,37].

### 4.2. Adverse Impacts of COVID-19 and Underlying Mechanisms of Its Influence on Mental Health

We found that the COVID-19 outbreak could significantly reduce young adults’ sleep quality and thereby increase their global negative emotions, especially stress and anxiety. This finding was consistent with a previous study revealing that individuals with better sleep quality or lower frequency of early awakenings showed reduced morbidity rates of PTSD during the COVID-19 outbreak [36]. Thus, the COVID-19 outbreak demonstrates an indirect influence on young adults’ mental health, with sleep quality playing an important mediating role. Considering that all colleges in China have shut down and may stay closed until September, Chinese college students will stay at home and keep social distancing for a long time. Under such circumstances, they might adopt irregular lifestyles with poor sleep quality, stress, and anxiety over their academics or future employment [1], as well as loneliness due to the lack of communication [38]. However, unexpectedly, COVID-19 might significantly reduce aggressiveness levels. We propose that this phenomenon probably results from people beginning to realize the fragility of life and cherishing every moment.

### 4.3. The Fundamental Role of Exercise and Its Dose-Response Associations with Mental Health

Furthermore, regular exercise is a good treatment for poor mental health and may directly reduce negative emotions within a certain range. This finding is in accordance with previous studies showing that daily physical activity was associated with a lower risk of psychiatric distress, regardless of form and intensity [39,40], that could improve immunity and maintain mood stability [41]. Some indoor exercises have been recommended during the disease outbreak, such as high-intensity interval training (HIIT), which helps facilitate metabolism, and yoga or relaxation training, which helps promote sleep quality and calm the mood [18,19]. Furthermore, it should be noted that the gym may be a high-risk infection area due to crowding [42]. Thus, staying at home and maintaining ventilation are as important as regular exercise during the COVID-19 period [42]. More importantly, the relationship between physical activity and mental health is nonlinear. In our study, 2500 METs of weekly physical activity appeared to minimize negative emotions during the COVID-19 outbreak, which is equivalent to about 108 min of light, 80 min of moderate, or 45 min of vigorous physical activity every day. The recommended PA load during this special period is slightly higher than in previous studies, in which 60 min of moderate-to-vigorous PA every day is recommended to maintain physical and psychological heath [43]. This is probably because of the special period of the COVID-19 outbreak: People need additional PA to offset the psychological burden and negative emotions caused by the disease outbreak and social distancing.

### 4.4. Limitations

This study has several limitations. First, considering the physical distancing status and difficulty of collecting longitudinal survey data, the sample size in our longitudinal study was not large enough and the study was performed on a relatively small group of exclusively Chinese college students as respondents. This study population may not be representative of the overall mental distress pattern of ordinary people of different ages, education levels, and countries. However, the findings in this study might to some extent reflect the psychological problems and intervention needs of the public during the COVID-19 disease period. Second, the study participants were predominantly females, who might tend to exercise less and be more vulnerable to negative emotions than their male counterparts. Thus, the associations between the COVID-19 outbreak, physical activity, and psychological health may be different between females and males. In addition, the preferred methods of coping with negative emotions, such as stress, anxiety, and depression, are different in females and males. Females may prefer to handle psychological distress based on avoidance and looking for social contacts. Therefore, further studies with larger samples are needed to explore the differences in the effectiveness of different forms of physical activity (e.g., group video exercise or interesting online sports) by gender. Third, we mainly focused on the impact of the local increases in COVID-19 death counts on the negative emotions of college students in social isolation status, and we did not exclusively focus on the effects of lengthened social distancing time. However, it was possible that the relationship between the results of psychological tests and the number of COVID-19 death counts might reflect the relationship between negative emotions and lengthened social distancing times. Hence, more studies are needed in the future to analyze the main impact of social distancing and the interactive effects of social isolation and the COVID-19 outbreak on public mental health. Furthermore, due to the physical distancing status across the whole country, we only used the validated self-reported DASS-21 questionnaire to measure the adverse impact of COVID-19 severity on negative emotions without having psychological consultants assess their mental health status as a form of third-party verification. This limitation might have resulted in self-report bias, but the results are generally reliable and consistent with the previous applications of DASS-21 in SARS-related and MERS-related studies. Moreover, due to restrictions of sample size, we did not compare the differences in variables between serious COVID-19 disease areas (e.g., inside Hubei Province) and non-serious COVID-19 disease areas (e.g., outside Hubei Province). We hope that other researchers from different countries or different areas will collect data in the future to analyze the regional differences in the impact of the COVID-19 on public mental health.

### 4.5. Strengths

This study also has several strengths. First, this study conducted longitudinal surveys to assess the adverse impact of the COVID-19 outbreak on Chinese college students’ mental health, sleep quality, and aggressiveness during the peak epidemic time. In addition, unlike cross-sectional studies, this longitudinal study used a mixed-effect model with random effects on individuals to control for inter-person variation. To the best of our knowledge, this study is the first to investigate the underlying mechanism whereby COVID-19 influences public mental health. Most importantly, our study found mitigation effects of regular exercise and good sleep on the public’s negative emotions without additional social medical burden. In addition, our study advances a suitable range of physical activity to maintain psychological health of 108 min of light, 80 min of moderate, or 45 min of vigorous physical activity daily, which we recommended for psychological interventions during the COVID-19 period.

## 5. Conclusions

In summary, during the peak phase of the COVID-19 outbreak in China, the severity of the COVID-19 outbreak has had an indirect effect on local young adults’ negative emotions, with sleep quality playing a mediating role. To deal with negative emotions during the disease outbreak, increasing physical activity and developing good sleeping patterns are cost-effective and practical mitigation strategies for ordinary people who are forced to stay at home. Developing a regular exercise habit with approximately 2500 METs of weekly physical activity is recommended during this special period. In addition, the COVID-19 outbreak has not had uniformly negative effects on public mental health, as the disease outbreak has reduced people’s aggressiveness levels, probably by making people realize the fragility of life and to treasure every day. Our findings could be used by policymakers or healthcare professionals to formulate public psychological interventions during the COVID-19 pandemic, which is, after all, still spreading and increasing worldwide.

## Figures and Tables

**Table 1:** Demographic characteristics and health status of all participants at baseline.

	Total	Male	Female	*p*-Value
***N***	66	25	41	
**Age (Year), Mean (*SD*)**	20.70 (2.11)	21.08 (1.94)	20.46 (2.19)	0.251
**Ethnicity, *n* (%)**				
Han	61 (92.42%)	24 (96.00%)	37 (90.24%)	0.367
Minority	5 (7.58%)	1 (4.00%)	4 (9.76%)	
**Body Mass Index, Mean (*SD*)**				
BMI (kg/m^2^)	21.11 (2.92)	22.54 (2.56)	20.24 (2.80)	0.001
**Residence, *n* (%)**				
City	51 (77.27%)	18 (72.00%)	33 (80.49%)	0.307
Countryside	15 (22.73%)	7 (28.00%)	8 (19.51%)	
**METs of Weekly PA, Mean (*SD*)**				
Vigorous PA	354.55 (613.41)	715.20 (785.62)	134.63 (367.78)	<0.001
Moderate PA	250.30 (374.19)	252.00 (301.66)	249.27 (415.84)	0.977
Light PA	327.50 (312.91)	462.00 (370.67)	245.49 (241.80)	0.005
Total PA	932.35 (881.69)	1429.200 (835.56)	629.390 (772.00)	<0.001
**Sedentary Behavior, Mean (*SD*)**				
Total minutes of weekly sedentariness	2881.06 (1086.27)	2830.40 (1351.02)	2911.95 (905.38)	0.770
**Physical Activity Level, *n* (%)**				
Sedentary	10 (15.15%)	2 (8.00%)	8 (19.51%)	0.002
Low	19 (28.79%)	2 (8.00%)	17 (41.46%)	
Moderate	29 (43.94%)	15 (60.00%)	14 (34.15%)	
High	8 (12.12%)	6 (24.00%)	2 (4.88%)	
**PSQI Level, *n* (%)**				
Healthy (global PSQI ≤ 5)	38 (57.58%)	14 (56.00%)	24 (58.54%)	0.520
Unhealthy (global PSQI > 5)	28 (42.42%)	11 (44.00%)	17 (41.46%)	
**Attitudes Towards COVID-19, *n* (%)**				
Worried or very concerned	56 (84.85%)	21 (84.00%)	35 (85.40%)	0.572
Not very worried or no care	10 (15.15%)	4 (16.00%)	6 (14.60)	
**Stress Level, *n* (%)**				
normal	47 (71.21%)	20 (80.00%)	27 (65.85%)	0.171
abnormal	19 (28.79%)	5 (20.00%)	14 (34.15%)	
**Anxiety Level, *n* (%)**				
normal	36 (54.55%)	17 (68.00%)	19 (46.34%)	0.072
abnormal	30 (45.45%)	8 (32.00%)	22 (53.66%)	
**Depression Level, *n* (%)**				
normal	51 (77.27%)	21 (84.00%)	30 (73.17%)	0.240
abnormal	15 (22.73%)	4 (16.00%)	11 (26.83%)	

Note: *N* means the number of participants who participated in this study. *n* indicates the number of people who met a certain characteristic of demographic variable or health status. *SD* means standard deviation.

**Table 2:** Relationship between COVID-19 death count and physical activity, sleep quality, and negative emotions.

	Every 1000 Increase in COVID-19 Death Cases		Every 100-MET Increase in Physical Activity	
	Point Estimate	95% CI	Point Estimate	95% CI
Sleep quality score	0.15	(−0.11, 0.41)	0.0015	(−0.0074, 0.010)
Sleep onset latency score	−0.07	(−0.24, 0.09)	0.0011	(−0.0058, 0.0081)
Sleep duration score	0.07	(−0.13, 0.26)	0.0026	(−0.0046, 0.0098)
Sleep efficiency score	0.29 *	(0.15, 0.44)	0.0001	(−0.0064, 0.0066)
Sleep disturbance score	0.16	(−0.08, 0.40)	0.0026	(−0.0075, 0.013)
Sleep medicine use score	−0.0002	(−0.02, 0.02)	−0.0001	(−0.0010, 0.00080)
Daytime dysfunction	0.12	(−0.04, 0.29)	0.0054	(−0.0015, 0.012)
Global PSQI score	1.37 *	(0.55, 2.19)	0.0110	(−0.022, 0.044)
Stress score	−0.66	(−2.09, 0.77)	−0.0389	(−0.085, 0.0068)
Anxiety score	0.18	(−0.95, 1.31)	−0.03	(−0.067, 0.0026)
Depression score	−0.03	(−1.33, 1.26)	−0.04 *	(−0.080, −0.0022)
Global DASS score	−0.01	(−3.54, 3.52)	−0.12 *	(−0.22, −0.010)
Aggressiveness score	−6.57 *	(−12.78, −0.36)	−0.03	(−0.17, 0.12)

Note: The point estimate indicates the change in each item for every 1000 increase in COVID-19 death cases or every 100-MET increase in physical activity. For example, for every 1000 increase in COVID-19 death cases, there is an associated 0.15 (95% confidence interval [95% CI]: −0.11, 0.41) increase in sleep quality score. A star (*) indicates significant effect of the COVID-19 or physical activity on outcome variables, e.g., Global PSQI score, at the 5% level.
